# Unmet Need for Family Planning: Implication for Under-five Mortality in Nigeria

**Published:** 2015-03

**Authors:** Sunday Adepoju Adedini, Clifford Odimegwu, Eunice Ntwala Imasiku, Dorothy Ngozi Ononokpono

**Affiliations:** ^1^Demography and Population Studies Programme, Schools of Public Health and Social Sciences, University of the Witwatersrand, Johannesburg, South Africa; ^2^Demography and Social Statistics Department, Obafemi Awolowo University, Ile-Ife, Nigeria; ^3^Department of Geography, University of Zambia, Lusaka, Zambia; ^4^Department of Sociology and Anthropology, University of Uyo, Uyo, Nigeria

**Keywords:** Contraception, Family planning, Under-five mortality, Unmet need, Nigeria

## Abstract

There are gaps in evidence on whether unmet need for family planning has any implication for under-five mortality in Nigeria. This study utilized 2008 Nigeria Demographic and Health Survey data to examine the effect of unmet need on under-five mortality. Cox regression analysis was performed on 28,647 children born by a nationally-representative sample of 18,028 women within the five years preceding the survey. Findings indicated elevated risks of under-five death for children whose mothers had unmet need for spacing [Hazard ratio (HR): 1.60, confidence interval (CI) 1.37-1.86, p<0.001] and children whose mothers had unmet need for limiting (HR: 1.78, CI 1.48-2.15, p<0.001) compared to children whose mothers had met need. These findings were consistent after adjusting for the effects of factors that could confound the association. Findings of this study underscore the need to address the present level of unmet need for family planning in Nigeria, if the country would achieve meaningful reduction in under-five mortality.

## INTRODUCTION

Despite the progress made in ensuring access to modern contraceptive methods in the recent time, women have continued to report an unmet need for family planning in the developing countries (1). While numerous women across the developing world would like to space or limit the number of their children, non-use of contraceptives is substantially high among them despite their sexual exposure and an expressed intention to avoid pregnancy (2). Unmet need for contraception is high in most parts of the developing world (3) as a result of low contraceptive-use (4). An estimated 105.2 million married women had an unmet need for family planning in the developing world (5) and, recently, the figure has increased to over 200 million (6).

The importance of contraceptive-use has been well-stressed in the demographic and public health literature, and several studies have documented the consequences of low or non-use of contraceptives. For instance, high levels of fertility have been linked with low contraceptive-use (7). Bankole and Ezeh (8) opined that fertility level has remained high in sub-Saharan Africa due to failure of family planning services and high unmet need in the region. In addition, issue of poverty has been linked with high fertility, particularly in countries with limited opportunities and high unemployment rates (3). Another consequence of excessive population growth arising from low contraceptive-use is the threat to environmental sustainability. Also, rapid population growth has been seen as having a great public-health consequence, particularly for maternal and child health. For instance, Cleland *et al.* projected that increasing contraceptive-use in countries with high fertility rates has the potential of averting about 32% of all maternal deaths and 10% of childhood deaths (3).

While unmet need for modern contraception is regarded as marginal in the middle-income countries (9), it remains unprecedentedly high in most of the sub-Saharan African countries. Especifically in Nigeria, Oye-Adeniran and colleagues (10) found that contraceptive prevalence (for both modern and traditional methods) among sexually-active women was 14.8%; most commonly-cited reasons for non-use of contraceptives were religious prohibition and fear of side-effects. Avong (11) argued that religious prohibition is an important factor to be addressed in government's effort to increase contraceptive-use and reduce family-size in Nigeria. Onwuzurike and Uzochukwu (12) argued that rejection by husband was the commonest single reason for non-use of contraceptives in Nigeria. Duze and Mohammed (13) corroborated this assertion by establishing that husbands’ willingness to allow their spouses to use contraceptives will determine the extent or pace of fertility reduction in Nigeria. Bongaarts and Bruce (14) suggest that there is need to address the cultural and familial factors that hinder the success of family planning services. While many studies have been conducted on unmet need for family planning and its consequences in Nigeria, there are gaps in evidence on whether unmet need for family planning has any implication for under-five mortality in Nigeria.

Further, considering child survival, around 7 million global under-five deaths were recorded in 2011 (15). With 41% of these deaths occurring in the sub-Saharan Africa (16), the region is the largest contributor to the statistics on childhood mortality. Specifically, the rate of under-five mortality stood at 157 per 1,000 livebirths in Nigeria. Like many other countries in the sub-Saharan Africa, Nigeria is not making sufficient progress towards the attainment of Millennium Development Goal 4 (MDG 4) to reduce under-five mortality (17).

Considering the fact that under-five mortality remains a major public-health challenge in Nigeria and other sub-Saharan African countries, several studies have shown the influence of various factors driving the phenomenon (18–27). While several of these studies have established a significant association between under-five mortality and various socioeconomic and biodemographic characteristics, similar studies on the relationship between unmet need and under-five mortality have been non-existent. Considering the high rate of under-five mortality in Nigeria (i.e. 157 per 1,000 livebirths) as well as the high unmet need for contraception (20%), it is important to examine whether unmet need for family planning has any implication for under-five mortality in Nigeria (28). Evidence shows that short birth interval, an index of unmet need for family planning, has implication for higher childhood mortality (28). Considering that unmet need for family planning has implication for short birth intervals and higher fertility levels, particularly through the mechanisms of too many births and too frequent births, we hypothesize that unmet need for family planning could lead to increased risks of under-five mortality. Hence, this paper examines the influence of unmet need for family planning on under-five mortality in Nigeria.

## MATERIALS AND METHODS

This study utilizes the 2008 Nigeria Demographic and Health Survey (NDHS) data. The survey was conducted in 2008 to elicit information on demographic and health indicators at the national and state levels. The primary sampling unit (PSU), which was regarded as a cluster for the 2008 NDHS, is defined on the basis of Enumeration Areas (EAs). Sample for the survey was selected using stratified two-stage cluster design consisting of 888 clusters (28). Data were collected by face-to-face interviews from the selected men and women. Data on women were used in the present analysis, and our analysis was child-based (i.e. we analyzed the birth recode of the 2008 NDHS). To obtain findings that are recent, analysis was restricted to births in the last five years before the survey. Out of the survey's complete sample-size of 33,385 women, the sample-size for this present study comprised 18,028 women who had a total of 28,647 livebirths within the five years preceding the survey. DHS programme elicited birth history, such as child's sex, birth order, month and year of birth as well as age at death if the child had died. Also, reproductive health-related data on contraceptive-use and unmet need for family planning were collected from the respondents. Detailed reports on methods used in the 2008 NDHS are available elsewhere (28).

### Ethical considerations

This paper is extracted from a larger study for which permission was granted for the use of 2008 NDHS data by ICF Macro International, USA. The study is based on secondary analysis of an existing survey data with all respondents’ identifiers information already removed. The ethical approval for the survey was granted by the Ethics Committee of the ICF Macro at Calverton in the United States and by the National Ethics Committee in Nigeria. Confidentiality and anonymity of all study participants were guaranteed.

### Measurement of variables

#### Outcome variable

In this study, the outcome variable is the risk of death before the age of five years, defined as the probability of dying before the fifth birthday. This is simply regarded as under-five mortality. Globally, under-five mortality is regarded as a good measure of wellbeing and socioeconomic development of a nation.

#### Explanatory variables

In terms of exposure, the main explanatory variable was unmet need for family planning. In the DHS data, fecund women who intend to delay (spacing) or stop childbearing (limiting) but have not been using any contraceptive method are regarded as having an unmet need for family planning. This variable was categorized as: (i) had met need, (ii) had unmet need for spacing, and (iii) had unmet need for limiting. Analysis was restricted to the birth history of women who reported either met need or unmet need (for limiting or spacing) in the five years before the survey. Birth history of all other categories of women, such as infecund and menopausal women, was dropped from the analysis.

In addition, of particular importance to this study are other independent variables capable of influencing or confounding the association between the outcome variable (under-five mortality) and the key explanatory variable (unmet need for family planning). These variables include: (i) number of living children, (ii) birth interval, (iii) birth order, (iv) place of delivery, (v) child's size at birth, (vi) mother's age at birth of the child, (vii) mother's current age, (viii) maternal education, (ix) mother's occupation, (x) household wealth index, (xi) religious affiliation, (xii) place of residence, (xiii) region of residence, and (xiv) ethnic affiliation.

### Statistical analysis

Three levels of analysis (univariate, bivariate, and multivariate) were employed in this study. At the univariate level, percentage distribution of respondents was presented to show the distribution of respondents by the selected variables. At the bivariate level, Pearson's chi-square test was performed to examine statistically significant relationship between the outcome variable and each of the selected explanatory variables. At the multivariate level, Cox proportional regression analysis was performed to explore the relationship between the dependent variable and a set of selected independent variables.

### Survival analysis

Cox proportional hazards model (survival analysis) is appropriate in analyzing non-censored and censored observations. Using Cox proportional hazards regression analysis, both occurrence of childhood mortality and the time when the child died were combined to generate the outcome variable. The children's survival status and the age at death in months (if the child had died) or the last month the child was known to be alive (if the child was still living at the time of the survey) were combined to generate the outcome variable for the survival analysis. Children known to have died before 60 months of age (i.e. non-censored) were regarded as the cases while children who were still alive at the time of the survey were treated as right-censored observations.

The probability of childhood death is regarded as the hazard. The hazard was modelled using the following equations:


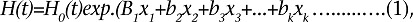


where X_1_ … X_k_ are a collection of explanatory variables and H_0_(t) is the baseline hazard at time t, representing the hazard for a person with the value 0 for all the explanatory variables. However, by dividing both sides of equation 1 by H_0_(t) and taking logarithms, the equation 1 becomes:


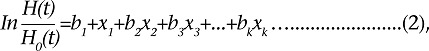


where H(t)/H_0_(t) is regarded as the hazard ratio. The coefficients b_i_…b_k_ are estimated by Cox regression.

Nine models were fitted at the multivariate level of analysis. While Model 1 presents the univariate hazard ratios (i.e. unadjusted hazard ratios), Model 2 to 9 present the multivariate or adjusted hazard ratios. Measures of association between outcome variable and explanatory variables were expressed as hazard ratios (HR) and 95% confidence interval. All analyses were done using Stata (version 11.2).

## RESULTS

### Descriptive analysis

Table 1 presents the description of the study sample. The study sample consisted of 51% males and 49% female children. The results showed that the largest group of the study sample were: children of women who had 5 or more living children (41.3%), children whose mothers were aged 18-34 years at birth of the child (77.4%), children born after birth interval of two or more years (80.8%), children of birth order 2-4 (46.2%), and children whose mothers were aged 25-34 years at the time of the survey (49.8%). Almost half of the children (46.5%) were children of mothers with no formal education while only 5% were children of mothers who had tertiary education. Nearly one-third (30.4%) were children of unemployed mothers while 3.7% were children of women in formal employment (i.e. professional/technical/managerial work). The highest proportion of the children was from the poorest households (23.2%), and the lowest was from the richest households (16.9%).

**Table 1.** Percentage distributions of study sample by selected demographic and socioeconomic characteristics

Variable/Category	Percentage	Number
Sex of child		
Male	50.9	14,604
Female	49.1	14,043
Number of living children		
<3	27.8	7,957
3-4	30.9	8,863
5+	41.3	11,827
Birth interval		
Less than 2 years	19.2	5,375
2 years or more	80.8	23,272
Birth order		
1	19.1	5,353
2-4	46.2	13,069
5+	34.7	10,225
Mother's age (years) at birth of the child		
<18	7.3	2,160
18-34	77.4	22,055
35+	15.3	4,432
Mother's current age (completed years)		
15-24	24.8	7,249
25-34	49.8	14,111
35+	25.4	7,287
Maternal education		
No formal education	46.5	14,418
Primary	23.2	6,552
Secondary	24.9	6,338
Higher	5.4	1,339
Mother's occupation		
Not working	30.4	9,035
Professional/Technical/Managerial	3.7	959
Clerical/Sales/Service	33.7	9,026
Manual labour	32.2	9,471
Household wealth index		
Poorest	23.2	7,604
Poorer	22.8	6,871
Middle	19.3	5,609
Richer	17.8	4,755
Richest	16.9	3,808
Religious affiliation		
Catholic	9.3	2,452
Other Christians	33.7	9,286
Muslim	55.4	16,152
Other religions	1.6	547
Mother's marital status		
Never married	1.7	506
Currently married	96.8	27,378
Previously married	1.6	446
Place of residence		
Urban	29.8	7,613
Rural	70.3	21,034
Region of residence		
South-West	11.6	3,318
North-Central	17.6	5,046
North-East	22.9	6,559
North-West	27.7	7,947
South-East	8.6	2,450
South-South	11.6	3,327
Met/Unmet need for family planning		
Had met need	35.3	3,695
Had unmet need for spacing	48.7	5,083
Had unmet need for limiting	16.0	1,669

With respect to mother's religious affiliation, the results showed that majority of the children (55.4%) were those of Muslim mothers. Another 9.3% were children of Catholic mothers while one-third were children of Christians other than Catholic. An overwhelmingly high proportion of children (96.8%) were those of the currently-married women. Seven out of 10 children (70.3%) were from rural areas. Considering region of residence, results showed that the highest proportion of children was from the North-West (27.7%), followed by children in the North-East (22.9%), North-Central (17.6%), South-West and South-South (11.6% each), and South-East (8.6%). With respect to the key explanatory variable (i.e. unmet need for family planning), Table 1 shows that majority of the study sample consisted of children whose mothers had unmet need for spacing (48.7%).

### Results of bivariate analysis

#### Characteristics of children and women by unmet need for family planning

Table 2 presents the distribution of the study sample by selected background characteristics and according to mothers’ situation of unmet need for family planning. The results showed that the percentage of children whose mothers reported unmet need for spacing was the highest for children of women who had 5 or higher parities (39.6%), children of uneducated women (49.0%), children of the unemployed (31.9%), children in the poorer households (22.8%), children of the Muslims (59.4%), children in the rural areas (72.0%), children of Hausa/Fulani/Kanuri tribes (43.8%), and children in the North-West region of the country (35.8%). In the same vein, the percentage of children whose mothers had unmet need for limiting was the highest for children of the uneducated women (35.8%), children of women who had 5 or higher parity (74.4%), children of manual labourer (38.1%), children of the Christians (45.4%), children in rural areas (69.2%), children of the minority ethnic groups (45.7%), and children in the South-West region (23.2%). Also, as shown in Table 2, women aged 25-34 years were more likely to report unmet need for spacing while those aged 35 years or older were more likely to report unmet need for limiting.

**Table 2.** Percentage distribution of children by unmet need for family planning (FP) according to selected characteristics of mother and child

Variable/Category	Had met need (%)	Unmet need	
		Had unmet need for spacing (%)	Had unmet need for limiting (%)
Unmet need for FP	35.3	48.7	16.0
Number of living children			
<3	28.3	27.4	8.1
3-4	38.4	33.0	17.5
5+	33.3	39.6	74.4
Birth interval			
Less than 2 years	19.3	21.5	19.3
2 years or more	80.7	78.5	80.7
Birth order			
1	19.4	18.7	5.7
2-4	53.2	47.2	25.5
5+	27.4	34.1	68.8
Place of delivery			
Home	30.1	67.2	55.5
Hospital	69.9	32.8	44.5
Child's size at birth			
Very large/Larger than average	51.1	46.8	54.7
Average	37.6	39.5	33.4
Very small/Smaller than average	11.3	13.7	11.9
Mother's age (years) at birth of the child			
<18	3.0	7.0	2.0
18-34	82.1	80.2	54.3
35+	14.9	12.8	43.7
Mother's current age (completed years)			
15-24	15.8	26.1	8.4
25-34	56.5	51.6	32.1
35+	27.7	22.3	59.5
Maternal education			
No formal education	11.3	49.0	35.8
Primary	25.7	23.2	32.8
Secondary	47.0	24.3	26.7
Higher	15.9	3.5	4.7
Mother's occupation			
Not working	20.2	31.9	21.7
Professional/Technical/Managerial	9.8	3.1	4.0
Clerical/Sales/Service	38.9	33.7	36.2
Manual labour	31.1	31.3	38.1
Household wealth index			
Poorest	6.1	22.2	16.5
Poorer	8.9	22.8	21.7
Middle	14.7	19.9	23.8
Richer	25.7	19.4	20.9
Richest	44.6	15.7	17.1
Religious affiliation			
Catholic	15.5	7.7	12.6
Other Christians	58.9	31.5	45.4
Muslim	24.5	59.4	40.4
Other religions	1.1	1.3	1.7
Place of residence			
Urban	54.0	28.0	30.8
Rural	46.0	72.0	69.2
Region of residence			
South-West	35.1	13.8	23.2
North-Central	11.7	12.0	13.5
North-East	5.3	15.4	12.8
North-West	5.9	35.8	19.2
South-East	17.2	7.6	11.9
South-South	24.8	15.4	19.4
Ethnic group			
Hausa/Fulani/Kanuri	6.2	43.8	20.3
Igbo	24.2	9.8	15.1
Yoruba	30.0	11.4	18.9
Others	39.6	34.9	45.7

With respect to characteristics of selected children, the proportion of children whose mothers had unmet need for spacing was the highest for children of birth order 2-4 (47.2%) and children whose place of delivery was home (67.2%). Similarly, percentage of children whose mothers had unmet need for limiting was the highest for children of the 5th or higher birth order (68.8%) and for children delivered at home (55.5%).

#### Under-five mortality, unmet need for family planning, and background characteristics

Table 3 presents the bivariate relationship between under-five mortality and the selected background characteristics. The results indicate that, with the expectation of mother's occupation, all selected characteristics were significantly associated with under-five mortality (p<0.05). Children whose mothers reported unmet need for spacing (10.2%) and unmet need for limiting (11.9%) were more likely to die before the age of five years compared to children whose mothers had met need (6.3%). Proportion of children who died before the age of five years was higher for children who had shorter preceding birth interval compared to those who had longer birth interval (13.1% vs 7.8%). Percentage of under-five death for children of the 5th or higher birth order was almost double that of children of the first order birth (12.0% vs 6.6%). Women who had the highest proportion of under-five death were also women with high parity. For instance, percentage of under-five deaths for women who had five or more living children was 12.4% against 4.9% for women who had 2 children or less.

**Table 3.** Percentage distribution of under-five children by their survival status and according to selected socioeconomic and demographic characteristics

Variable/Category	Child's survival status		(Chi-square) p value
	Alive (N=25,446)	Dead (N=3,201)	
Met/Unmet need for contraception			(23.2) 0.00
Had unmet need for spacing	93.7	6.3	
Had unmet need for limiting	89.8	10.2	
Had met need	88.1	11.9	
Birth interval			(56.9) 0.00
Less than 2 years	86.9	13.1	
2 years or more	92.2	7.8	
Birth order			(30.1) 0.00
1	93.4	6.6	
2-4	92.7	7.3	
5+	88.0	12.0	
Child's size at birth			(11.4) 0.00
Very large/Larger than average	92.7	7.3	
Average	91.7	8.3	
Very small/Smaller than average	88.2	11.8	
Number of living children			(50.3) 0.00
<3	95.1	4.9	
3-4	92.6	7.4	
5+	87.6	12.4	
Place of delivery			(37.4) 0.00
Home	89.4	10.6	
Health facility	93.4	6.6	
Maternal education			(19.2) 0.00
No formal education	88.0	12.0	
Primary	91.0	9.0	
Secondary	92.8	7.2	
Higher	96.3	3.7	
Mother's occupation			(2.5) 0.058
Not working	90.6	9.4	
Professional/Technical/Managerial	94.3	5.7	
Sales/Clerical/Service	91.5	8.5	
Manual labour	90.6	9.4	
Household wealth index			(17.4) 0.00
Poorest	87.4	12.6	
Poorer	87.9	12.1	
Middle	90.7	9.3	
Richer	91.8	8.2	
Richest	94.9	5.1	
Alive (N=25,446)	Dead (N=3,201)
Religious affiliation			(7.3) 0.00
Catholic	92.1	7.9	
Other Christians	92.4	7.6	
Muslim	89.5	10.5	
Others	92.7	7.3	
Ethnic affiliation			(16.8) 0.00
Hausa/Fulani/Kanuri	87.7	12.3	
Igbo	92.3	7.7	
Yoruba	94.9	5.1	
Others	91.0	9.0	
Mother's marital status			(3.1) 0.047
Never married	90.1	9.9	
Currently married	91.2	8.8	
Previously married	82.4	17.6	
Mother's age (years) at birth of the child			(16.2) 0.00
<18	87.1	12.9	
18-34	92.1	7.9	
35+	88.0	12.0	
Mother's current age (completed years)			(11.1) 0.00
15-24	91.0	9.0	
25-34	92.4	7.6	
35+	89.0	11.0	
Place of residence			(33.1) 0.00
Urban	93.6	6.4	
Rural	89.5	10.5	
Region of residence			(12.8) 0.00
South-West	94.7	5.3	
North-Central	91.4	8.6	
North-East	87.2	12.8	
North-West	88.1	11.9	
South-East	91.2	8.8	
South-South	92.1	7.9	

The results in Table 3 further show that proportion of under-five deaths was the highest for women who had no education (12.0%), unemployed women and those in the manual labour (9.4%), women in the poorest households (12.6%), Muslim women (10.5%), previously-married women (17.6%), rural women (10.5%), Hausa/Fulani/Kanuri women (12.3%), and women of 17 years or younger at the time of childbirth (12.9%).

In contrast, the percentage of children who died before the age of five years was the lowest for women who had tertiary education (3.7%), women in formal employment (5.7%), women in the richest wealth quintile (5.1%), Christian women (7.9%), urban women (6.4%), Yoruba women (5.1%), and women aged 18-34 years (7.9%). Considering region of residence, proportion of children who died before the age of five years was the highest for women residing in the North-East (12.8%) and North-West (11.9%) while it was the lowest for mothers residing in the South-West (5.3%).

### Results of multivariate survival analysis

**Figure 1.**
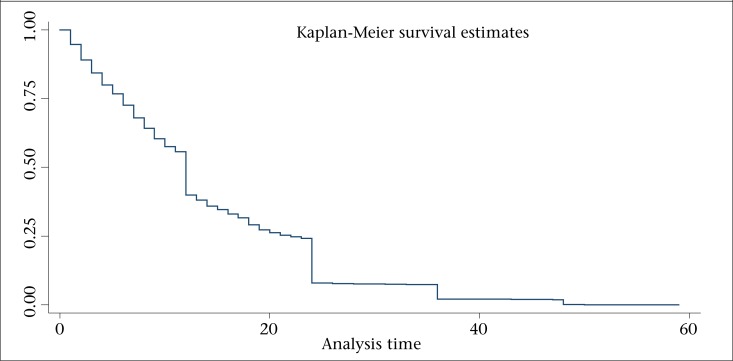
Child survival plot for children who died during the first five years of life

**Figure 2.**
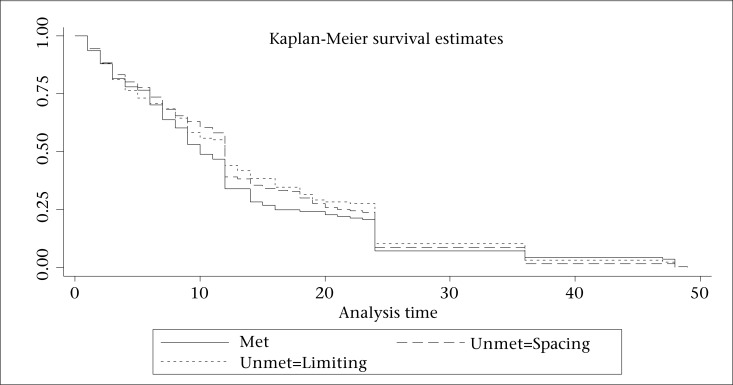
Child survival plots for children who died before the age of 60 months (by unmet need for family planning)

Figure 1 presents the child survival plot showing the duration of survival since birth for the non-censored cases [i.e. children who died during the first 5 years of life, within the age of 0-59 month(s)]. Further description of the mortality risks among those children who did not survive beyond the age of five years by unmet need for family planning is provided in Figure 2.

**Table 4.** Univariate hazard ratios and confidence interval showing the effects of unmet need for family planning and other selected characteristics on child survival, Nigeria 2008

Variable/Category	Model 1		
	Hazard ratios	Confidence interval	Significance level
Met/Unmet need for contraception		
Had unmet need for spacing	1		
Had unmet need for limiting	1.60	1.37-1.86	***
Had met need	1.78	1.48-2.15	***
Number of living children			
<3	1		
3-4	1.52	1.23-1.87	***
5+	2.48	2.05-3.00	***
Birth order			
1	1		
2-4	1.10	0.90-1.36	
5+	1.78	1.46-2.18	***
Birth interval			
Less than 24 months	1		
24 months or higher	0.57	0.50-0.66	***
Educational level			
No formal education	1		
Primary	0.76	0.65-0.89	***
Secondary	0.58	0.50-0.69	***
Tertiary	0.28	0.19-0.40	***
Mother's occupation			
Not working	1		
Professional/Technical/Managerial	0.57	0.40-0.82	**
Sales/Clerical/Service	0.87	0.74-1.03	
Manual labour	0.94	0.80-1.10	
Wealth index			
Poorest	1		
Poorer	0.95	0.80-1.14	
Middle	0.71	0.59-0.86	***
Richer	0.65	0.54-0.79	***
Richest	0.39	0.31-0.49	***
Religious affiliation			
Catholic	1		
Other Christians	1.02	0.81-1.29	
Muslim	1.40	1.11-1.75	**
Others	0.99	0.55-1.78	
Mother's marital status		
Never married	1		
Currently married	1.06	0.63-1.80	
Previously married	2.10	1.01-4.35	*
Mother's age (years) at birth of the child		
<18	1		
18-34	0.61	0.48-0.79	***
35+	0.90	0.69-1.18	
Mother's age (completed years)			
15-24	1		
25-34	0.89	0.75-1.06	
35+	1.24	1.04-1.48	*
Place of residence			
Urban	1		
Rural	1.60	1.38-1.85	***
Mothers’ ethnic affiliation			
Hausa/Fulani/Kanuri	1		
Igbo	0.65	0.52-0.80	***
Yoruba	0.40	0.31-0.51	***
Others	0.77	0.67-0.89	***
Region of residence			
South-West	1		
North-Central	1.56	1.21-2.01	**
North-East	2.43	1.92-3.09	***
North-West	2.21	1.75-2.79	***
South-East	1.71	1.30-2.26	***
South-South	1.46	1.13-1.89	***

*p<0.05

**p<0.01

***p<0.001

Results from the proportional hazards regression analysis are presented in Table 4. The results as shown in Model 1 (i.e. unadjusted hazard ratios) indicated a significant relationship between unmet need for family planning and under-five mortality. For instance, findings showed that the risks of under-five deaths were almost two-fold significantly higher for children whose mothers had unmet need for spacing (HR: 1.60, CI 1.37-1.86, p<0.001) and children whose mothers had unmet need for limiting (HR: 1.78, CI 1.48-2.15, p<0.001) compared to children whose mothers reported met need. Similarly, results in Table 4 reveal a significant association between child survival and some selected background characteristics. For instance, the risks of under-five death were significantly higher for children of the 5th or higher birth order (HR: 1.78, CI 1.48-2.18, p<0.001), children whose mothers had 5 or more living children (HR: 2.48, CI 2.05-3.00, p<0.001), children of Muslim mothers (HR: 1.40, CI 1.11-1.75, p<0.01), children of previously-married women (HR: 2.10, CI 1.01-4.35, p<0.05), children of mothers aged 35 years or older (HR: 1.24, CI 1.04-1.48, p<0.05), children of mothers residing in rural areas (HR: 1.60, CI 1.38-1.85, p<0.001); children in the North-Central (HR: 1.56, CI 1.21-2.01, p<0.01), North-East (HR: 2.43, CI 1.92-3.09, p<0.001), North-West (HR: 2.21, CI 1.75-2.79, p<0.001), South-East (HR: 1.71, CI 1.30-2.26, p<0.001), and South-South (HR: 1.46, CI 1.13-1.89, p<0.001) region compared to children in the reference categories.

Conversely, results in Table 4 reveal that the risks of dying before the age of five years were significantly lower for children born after preceding birth interval of 24 months or longer (HR: 0.57, CI 0.50-0.66, p<0.001), children of mothers who had tertiary education (HR: 0.28, CI 0.19-0.40, p<0.001), children whose mothers were engaged in professional/managerial/technical work (HR: 0.57, CI 0.40-0.82, p<0.01), children of women in the richest wealth quintile (HR: 0.39, CI 0.31-0.49, p<0.001), children of women aged 18-34 years at childbirth (HR: 0.61, CI 0.48-0.79, p<0.001), children of Igbo women (HR: 0.65, CI 0.52-0.80, p<0.001), and children of Yoruba women (HR: 0.40, CI 0.31-0.51, p<0.001).

Table 5 through 7 present the results of multivariate hazard ratios. The risk factors of under-five mortality by unmet need were controlled separately for children's and mothers’ characteristics. In Model 2 through 4 (Table 5), we examined the association between unmet need and child survival while adjusting for the effects of selected children's characteristics. In Model 5 through 7 (Table 6), we examined the association between unmet need and child survival, adjusting for women's characteristics. While Model 8 (Table 7) is the full model which incorporated the selected children and mothers’ characteristics, in Model 9—the final model (Table 7), we employed stepwise Cox regression analysis to determine the key variables influencing association between unmet need and child survival.

Thus, because birth interval and birth order were found to be correlated, albeit weakly (analysis not shown), we first incorporated each of these two variables into different models in Model 2 and 3. However, even after combining all the selected children's characteristics in Model 4, we found results similar to those found in Model 2 and 3.

As shown in Model 2-4 (Table 5), after adjusting for the effects of selected children's characteristics, unmet need for family planning remained significantly associated with under-five mortality. Results in Model 4, for instance, indicate significantly higher risks of dying before the age of five years for children whose mothers had unmet need for spacing (HR: 1.55, CI 1.32-1.82, p<0.001) and children whose mothers had unmet need for limiting (HR: 1.49, CI 1.21-1.83, p<0.001) compared to children whose mothers had no unmet need. Also, risks of death were almost five-fold significantly higher for women who had 5 or more living children (HR: 4.70, CI 3.21-6.90, p<0.001). Multivariate hazard ratios indicated significantly lower risks of under-five death for children of higher birth order (HR: 0.37, CI 0.24-0.56, p<0.001) compared to children of the first birth order, and for children with longer preceding birth interval (HR: 0.61, CI 0.53-0.71, p<0.001) compared to children who had shorter birth interval.

**Table 5.** Multivariate hazard ratios (HR) and confidence intervals (CIs) showing the effects of unmet need for family planning on child survival, adjusting for effects of selected children's characteristics, Nigeria 2008

Variable/Category	Model 2 HR (CI)	Model 3 HR (CI)	Model 4 HR (CI)
Met/Unmet need for contraception			
Had met need	1	1	1
Had unmet need for spacing	1.58 (1.34-1.85)***	1.55 (1.32-1.82)***	1.55 (1.32-1.82)***
Had unmet need for limiting	1.47 (1.20-1.81)***	1.46 (1.19-1.79)***	1.49 (1.21-1.83)***
Number of living children			
<3	1	1	1
3-4	2.37 (1.72-3.27)***	1.50 (1.20-1.88)***	2.39 (1.73-3.29)***
5+	4.80 (3.27-7.04)***	2.30 (1.87-2.83)***	4.70 (3.21-6.90)***
Child's size at birth			
Very large/Larger than average	1	1	1
Average	1.10 (0.95-1.28)	1.10 (0.95-1.27)	1.10 (0.95-1.27)
Very small/Smaller than average	1.48 (1.23-1.78)***	1.46 (1.22-1.76)***	1.46 (1.21-1.76)***
Birth order			
1	1		1
2-4	0.55 (0.39-0.77)***	---	0.48 (0.34-0.67)***
5+	0.41 (0.27-0.62)***	---	0.37 (0.24-0.56)***
Birth interval			
Less than 24 months	---	1	1
24 months or higher	---	0.64 (0.55-0.74)***	0.61 (0.53-0.71)***

***p<0.001

**Table 6.** Multivariate hazard ratios (HR) and confidence intervals (CIs) showing the effects of unmet need for family planning on child survival, adjusting for selected mother's characteristics, Nigeria 2008

Variable/Category		Model 5 HR (CI)	Model 6 HR (CI)	Model 7 HR (CI)
Met/Unmet need for contraception			
Had met need	1	1	1
Had unmet need for spacing	1.18 (0.99-1.40)	1.21 (1.02-1.44)*	1.18 (0.99-1.40)
Had unmet need for limiting	1.28 (1.04-1.58)*	1.31 (1.06-1.61)*	1.30 (1.05-1.59)*
Educational level			
No formal education	1	-	1
Primary	0.96 (0.80-1.16)	-	0.98 (0.81-1.18)
Secondary	0.89 (0.71-1.12)	-	0.87 (0.70-1.10)
Tertiary	0.50 (0.32-0.78)**	-	0.45 (0.27-0.73)**
Mother's occupation			
Not working	-	1	1
Professional/Technical/Managerial	-	0.91 (0.63-1.33)	1.29 (0.85-1.95)
Sales/Clerical/Service	-	1.01 (0.85-1.19)	1.00 (0.85-1.19)
Manual labour	-	1.00 (0.84-1.18)	0.98 (0.82-1.16)
Wealth index			
Poorest	1	1	1
Poorer	1.05 (0.87-1.26)	1.05 (0.87-1.26)	1.05 (0.87-1.27)
Middle	1.05 (0.72-1.10)	0.89 (0.73-1.10)	0.90 (0.73-1.12)
Richer	0.95 (0.75-1.20)	0.92 (0.73-1.16)	0.95 (0.75-1.21)
Richest	0.74 (0.55-1.01)	0.65 (0.49-0.88)**	0.75 (0.55-1.02)
Religious affiliation			
Catholic	1	1	1
Other Christians	1.14 (0.89-1.46)	1.15 (0.89-1.48)	1.13 (0.88-1.45)
Muslim	1.14 (0.84-1.55)	1.21 (0.90-1.64)	1.13 (0.83-1.54)
Others	0.80 (0.44-1.44)	0.87 (0.48-1.57)	0.82 (0.45-1.48)
Mother's marital status		
Never married	1	1	1
Currently married	0.86 (0.50-1.48)	0.88 (0.51-1.52)	0.89 (0.52-1.54)
Previously married	1.89 (0.89-4.01)	1.98 (0.94-1.19)	1.98 (0.94-4.18)
Mother's age (years) at birth of the child		
<18	1	1	1
18-34	0.66 (0.49-0.90)*	0.67 (0.50-0.91)*	0.68 (0.50-0.92)*
35+	0.80 (0.54-1.16)	0.81 (0.55-1.18)	0.80 (0.55-1.18)
Mother's current age (completed years)			
15-24	1	1	1
25-34	1.14 (0.93-1.41)	1.13 (0.92-1.39)	1.13 (0.92-1.40)
35+	1.26 (0.97-1.64)	1.26 (0.97-1.64)	1.26 (0.97-1.64)
Place of residence			
Urban	1	1	1
Rural	1.16 (0.97-1.39)	1.19 (1.00-1.43)	1.20 (1.00-1.43)*
Mothers’ ethnic affiliation			
Hausa/Fulani/Kanuri	1	1	1
Igbo	0.79 (0.48-1.29)	0.76 (0.46-1.03)	0.80 (0.49-1.31)
Yoruba	0.72 (0.48-1.07)	0.69 (0.46-1.03)	0.73 (0.47-1.09)
Others	0.95 (0.75-1.19)	0.95 (0.75-1.19	0.96 (0.76-1.20)
Region of residence			
South-West	1	1	1
North-Central	1.06 (0.75-1.51)	1.04 (0.73-1.48)	1.05 (0.74-1.50)
North-East	1.38 (0.95-2.01)	1.35 (0.93-1.96)	1.38 (0.95-2.01)
North-West	1.24 (0.84-1.83)	1.20 (0.82-1.78)	1.23 (0.83-1.82)
South-East	1.69 (1.01-2.84)*	1.67 (0.99-2.8)	1.65 (0.98-2.77)
South-South	1.12 (0.78-1.62)	1.09 (0.76-1.58	1.11 (0.77-1.60)

*p<0.05

**p<0.01

In addition, mother's education and mother's occupation were found to be correlated, albeit weakly (because the level of education tends to determine the type of employment); we first included the two variables in different models before combining all the selected mother-level variables in Model 7. Findings in Model 5 and 6 are not much different from the results in Model 7 (Table 6). While Model 5 indicated significant relationships between under-five mortality and unmet need for limiting (HR: 1.28, CI 1.04-1.58, p<0.05), Model 6 (which excluded mother's education) indicated significant association between under-five mortality and unmet need for spacing (HR: 1.21, CI 1.02-1.44, p<0.05) and unmet need for limiting (HR: 1.31, CI 1.06-1.61, p<0.05). Model 7 (which considered all the selected women's characteristics) indicate a significantly higher risks of dying before the age of five years for children whose mothers had unmet need for limiting (HR: 1.30, CI 1.05-1.59, p<0.05) and children whose mothers were living in rural areas (HR: 1.20, CI 1.00-1.43, p<0.05).

The results of Model 8 which incorporated all the selected characteristics are presented in Table 7. As noted earlier, Model 9 was fitted to determine the key factors influencing the association between child survival and unmet need for family planning. Results from the stepwise Cox regression analysis as shown in Model 9 reveal that hazards of dying before the age of five years were significantly higher for children whose mothers had unmet need for spacing (HR: 1.23, CI 1.03-1.48, p<0.05) and children whose mothers had unmet need for limiting (HR: 1.26, CI 1.01-1.56, p<0.05).

Considering other significant covariates in Model 9, being a child of a mother who had 5 or more living children (HR: 5.58, CI 3.71-8.38, p<0.001), being a child whose size at birth was very small (HR: 1.33, CI 1.10-1.61, p<0.001), being a child of a rural woman (HR: 1.24, CI 1.03-1.50, p<0.05), being a child of a woman residing in North-East (HR: 1.49, CI 1.13-1.98, p<0.01); North-West (HR: 1.42, CI 1.08-1.87, p<0.05), and South-East (HR: 1.36, CI 1.01-1.85, p<0.05) were significantly associated with elevated risks of dying before the age of five years compared to children in the reference categories. Results in Model 9 further showed that risks of death were significantly lower for children of higher birth order (HR: 0.39, CI 0.25-0.61, p<0.001), children who had longer birth interval (HR: 0.63, CI 0.54-0.73, p<0.001), children of mothers in the richest wealth quintile (HR: 0.64, CI 0.47-0.86, p<0.01), and children of mothers aged 25-34 years (HR: 0.76, CI 0.60-0.97, p<0.05).

**Table 7.** Multivariate hazard ratios (HR) and confidence intervals (CIs) showing the effects of unmet need for family planning on child survival, adjusting for selected mothers’ and children's characteristics, Nigeria 2008

Variable/Category	Model 8 HR (CI)	Model 9 HR (CI)
Met/Unmet need for contraception		
Had met need	1	1
Had unmet need for spacing	1.20 (1.00-1.44)	1.23 (1.03-1.48)*
Had unmet need for limiting	1.24 (1.00-1.54)*	1.26 (1.01-1.56)*
Number of living children		
<3	1	1
3-4	2.68 (1.91-3.76)***	2.73 (1.95-3.82)***
5+	5.34 (3.55-8.09)***	5.58 (3.71-8.38)***
Child's size at birth		
Very large/Larger than average	1	1
Average	1.06 (0.91-1.23)	1.06 (0.91-1.23)
Very small/Smaller than average	1.34 (1.10-1.62)**	1.33 (1.10-1.61)**
Birth order		
1	1	1
2-4	0.58 (0.40-0.83)**	0.58 (0.41-0.84)**
5+	0.34 (0.25-0.61)***	0.39 (0.25-0.61)***
Birth interval		
Less than 24 months	1	1
24 months or higher	0.63 (0.54-0.73)***	0.63 (0.54-0.73)***
Educational level		
No formal education	1	
Primary	1.02 (0.83-1.23)	NS
Secondary	0.99 (0.78-1.27)	NS
Tertiary	0.52 (0.30-0.90)*	NS
Mother's occupation		
Not working	1	
Professional/Technical/Managerial	1.32 (0.85-2.06)	NS
Sales/Clerical/Service	0.97 (0.81-1.16)	NS
Manual labour	0.94 (0.79-1.13)	NS
Wealth index		
Poorest	1	1
Poorer	0.99 (0.81-1.20)	0.99 (0.81-1.20)
Middle	0.86 (0.69-1.07)	0.87 (0.70-1.08)
Richer	0.91 (0.70-1.16)	0.92 (0.72-1.16)
Richest	0.68 (0.49-0.94)*	0.64 (0.47-0.86)**
Religious affiliation		
Catholic	1	
Other Christians	1.10 (0.85-1.43)	NS
Muslim	1.04 (0.75-1.44)	NS
Others	0.76 (0.40-1.45)	NS
Mother's marital status		
Never married	1	1
Currently married	0.67 (0.37-1.20)	0.66 (0.37-1.17)
Previously married	1.72 (0.78-3.79)	1.67 (0.76-3.65)
Mother's age (years) at birth of the child		
<18	1	1
18-34	0.58 (0.41-0.81)**	0.57 (0.41-0.79)**
35+	0.73 (0.48-1.10)	0.72 (0.48-1.08)
Mother's current age (completed years)		
15-24	1	1
25-34	0.79 (0.62-1.01)	0.76 (0.60-0.97)*
35+	0.76 (0.55-1.04)	0.73 (0.53-1.00)
Place of residence		
Urban	1	1
Rural	1.24 (1.02-1.50)*	1.24 (1.03-1.50)*
Mothers’ ethnic affiliation		
Hausa/Fulani/Kanuri	1	
Igbo	0.72 (0.43-1.222)	NS
Yoruba	0.79 (0.52-1.22)	NS
Others	0.93 (0.73-1.18)	NS
Region of residence		
South-West	1	1
North-Central	1.07 (0.73-1.56)	1.17 (0.88-1.56)
North-East	1.31 (0.87-1.96)	1.49 (1.13-1.98)**
North-West	1.20 (0.79-1.82)	1.42 (1.08-1.87)*
South-East	1.57 (0.89-2.75)	1.36 (1.01-1.85)*
South-South	1.05 (0.71-1.56)	1.18 (0.89-1.57)

*p<0.05

**p<0.01

***p<0.001;

NS=Not selected

## DISCUSSION

We have examined the implication of unmet need for under-five mortality in Nigeria. The data were from livebirths in the five years before the survey among women who reported met need or unmet need during the period under study. After adjusting for the effects of important covariates (which could influence the relationship between unmet need and child survival), such as maternal education, number of living children, religious affiliation, mother's occupation, mother's marital status, place of residence, wealth index, mother's age at birth of the child, and region of residence, the results indicated a significant association between under-five mortality and unmet need for family planning in Nigeria.

Findings of this study suggest that higher risk of dying before the age of five years was associated with unmet need for family planning in Nigeria, thus lending credence to earlier study, which suggests that increase in contraceptive-use could bring about reduction in the rates of childhood mortality (3).

While establishing unmet need as an important predictor of child survival, this study also established maternal education as an important factor influencing child survival, thus supporting findings of earlier studies which established maternal education as an important factor influencing child survival (22,29–31). Amongst other reasons, uneducated women are likely to have little or no knowledge about the varieties of contraceptive methods available for their use. They are also likely to know little or nothing about the appropriate use of contraceptive methods. Inconsistent use of contraceptive methods could also be another problem faced by an uneducated woman. Hence, uneducated women are likely to have very close births and too many births, which could negatively affect the health of the children and of the mother.

As has been found previously (26,32–35), the present study established that children of mothers in rural areas were having significantly higher risk of dying before the age of five years compared to their counterparts in the urban centres. This is expected as women in rural areas tend to have limited access to contraceptive methods. Besides, rural women are more likely to be less educated (35); hence, they are more likely to have limited knowledge about the varieties of contraceptive methods available either to limit or space births. As a result of limited access, together with little or no knowledge about contraceptive methods in the rural areas, rural women are more likely to have very short birth intervals, thereby predisposing the child and the mother to poor health outcomes. Previous studies have established significant relationship between short birth interval and higher childhood mortality (36–38).

Further, results showed a significantly higher risk of death among children of mothers in the poor households. It could be reasonably assumed that even if poor women have knowledge about the contraceptive methods that are available for their use (either to limit or space births), it may be unaffordable to them. This is because contraceptive commodities are not absolutely free in most developing countries (39). The issue of old-age security may be another strong reason for many poor people to desire having so many children even if contraceptive commodities are available and affordable. This factor is a serious source of concern in Nigeria due to mismanagement of resources, corruption in governance, and failure of governments to improve living condition of people (40). Hence, people have little or nothing to save from their incomes. Many poor people would rather choose to have so many children in order to take care of them when they (parents) become old. Thus, as established in this study that having many children is associated with higher risks of under-five mortality, previous findings have shown that such higher levels of fertility are significantly associated with higher childhood mortality (41).

As found earlier by Antai *et al*. (42), this study found that children of Muslim mothers were having higher risk of dying before the age of five years compared to children of Christian mothers. This may be partly due to the fact that Muslim women tend to face oppositions regarding the use of contraceptive methods from their husbands (14). This is because the Islam religion particularly forbids using contraception to restrict the overall size of families (43). Further, mother's age at childbirth showed statistical significance for under-five mortality as children born to mothers aged 18-34 years and those aged 35 years or older were having lower risk of death than the children of women younger than 18 years. Plausible explanation for this could be that older women are more likely to have knowledge about the varieties of appropriate contraceptive methods as well as the financial means to afford them. In addition, findings established significantly higher risks of under-five mortality in other regions of the country compared to the South-West. This could be partly due to disparities in spatial inequality in social development across regions (44).

### Strengths and limitations

This study has its limitations. First limitation concerns the unobserved heterogeneity which could result from non-inclusion of some important factors in our analysis. These important factors (such as sociocultural practices which could influence unmet need and under-five mortality) could not be considered in this study. Being an analysis of a secondary dataset, such factors were not available in the DHS dataset. Second, being a cross-sectional study, a cause-effect relationship could not be established. Nonetheless, this study has its strengths also. The study used 2008 NDHS data from a nationally-representative dataset. Hence, findings of the study could be a true representation of the situation in the whole country. Also, because DHS surveys adopt similar instruments across countries, international comparisons of results are possible.

### Conclusions

The findings of this study established that unmet need for family planning has implication for increased risks of under-five mortality in Nigeria. The findings demonstrate the importance of contraceptive-use in the pursuit of the realization of childhood mortality reduction in Nigeria. The results of this study underscore the need to address the present level of unmet need for family planning if substantial reduction in under-five mortality is to be achieved in the country.

## ACKNOWLEDGEMENTS

The research received funding from a fellowship award provided by the Consortium for Advanced Research Training in Africa (CARTA). An earlier version of this paper was presented at the 2nd Population Conference of the Asian Population Association held in Bangkok, Thailand, from 26 to 29 August 2012. Authors are grateful for the comments received from the participants at the conference which helped improve the quality of this paper. Authors also wish to thank ICF Macro for permission to use the Nigeria DHS data. The support for writing retreat from the University of the Witwatersrand Strategic Planning and Resource Allocation Committee (SPARC) is also gratefully acknowledged.

**Conflict of interest:** Authors declare they had no conflicts of interest.
